# Synergistic effect of VEGF and SDF-1α in endothelial progenitor cells and vascular smooth muscle cells

**DOI:** 10.3389/fphar.2022.914347

**Published:** 2022-07-15

**Authors:** Haiyan Yang, Cancan He, Yang Bi, Xu Zhu, Dan Deng, Tingting Ran, Xiaojuan Ji

**Affiliations:** ^1^ Department of Ultrasound, Chongqing General Hospital, Chongqing, China; ^2^ Department of Macromolecular Science, State Key Laboratory of Molecular Engineering of Polymers, Fudan University, Shanghai, China; ^3^ Department of Pediatrics, The Affiliated Hospital of Zunyi Medical University, Guizhou Children’s Hospital, Zunyi, GZ, China; ^4^ Ministry of Education Key Laboratory of Child Development and Disorders, National Clinical and Research Center of Child Health and Disorders, Chongqing Engineering Research Center of Stem Cell Therapy, Department of Ultrasound, China International Science and Technology Cooperation Base of Child Development and Critical Disorders, Children’s Hospital of Chongqing Medical University, Chongqing, China; ^5^ School of Medical Imaging, Changsha Medical University, Changsha, China

**Keywords:** vascular smooth muscle cells, vascular endothelial growth factor, endothelial progenitor cell, stromal cell-derived factor-1α, synergistic effect

## Abstract

Vascular endothelial growth factor (VEGF) is a potent agonist of angiogenesis that induces proliferation and differentiation of endothelial progenitor cells (EPCs) after vascular injury. Previous studies have suggested that stromal cell-derived factor 1-alpha (SDF-1α) and VEGF have a synergistic effect on vascular stenosis. The aim of the present study was to investigate whether VEGF and SDF-1α act synergistically in EPCs and vascular smooth muscle cells (VSMCs). In this study, EPCs were isolated from rat bone marrow and their morphology and function were studied. Subsequently, VEGF was delivered into EPCs using an adenoviral vector. Tube formation, migration, proliferation, and apoptosis of VEGF-overexpressing EPCs was analyzed. Then, EPCs were co-cultured with VSMCs in the presence or absence of SDF-1α, the migration, proliferation, apoptosis, and differentiation capacity of EPCs and VSMCs were analyzed respectively. The isolated EPCs showed typical morphological features, phagocytic capacity, and expressed surface proteins. While stable expression of VEGF remarkably enhanced tube formation, migration, and proliferation capacity of EPCs, apoptosis was decreased. Moreover, the proliferation, migration, and differentiation capacity of EPCs in the co-cultured model was enhanced in the presence of SDF-1α, and apoptosis was decreased. However, these effects were reversed in VSMCs. Therefore, our results showed that VEGF and SDF-1α synergistically increased the migration, differentiation, and proliferation capabilities of EPCs, but not VSMCs. This study suggests a promising strategy to prevent vascular stenosis.

## Introduction

The increased incidence of congenital and acquired vascular stenosis is a serious concern for human health. Percutaneous endovascular angioplasty and stent implantation are routinely used for treatment of stenosis; however, these methods can cause restenosis after operation (Poon et al., 2002). The major mechanism underlying vascular stenosis is proliferation of vascular smooth muscle cells (VSMCs) and intimal hyperplasia caused by injury, which leads to vascular reconstruction and lumen stenosis (Chaabane et al., 2013). Treatment of vascular stenosis is primarily achieved using drugs, stents, and gene targeting therapies that inhibit proliferation and migration of VSMCs (Gray and Granada, 2010; Qiu et al., 2022). However, these methods are not very effective. Therefore, there is a need to develop non-invasive and effective methods to prevent and treat vascular stenosis that promote vascular endothelium repair and inhibit proliferation of VSMCs.

Endothelial progenitor cells (EPCs) were first isolated from adult peripheral blood in 1997 (Asahara et al., 1997). Accumulating evidence has indicated that EPCs play an important role in vascular endothelial repair by maintaining structural integrity and repair function after arterial injury; this is a post-natal vasculogenic process (Leu et al., 2016). Following vascular stenosis or endothelial damage, EPCs are mobilized from the bone marrow to vascular injury sites where they repair the damaged endothelium and improve neovascularization (Williamson et al., 2012). The therapeutic use of EPCs for vascular stenosis treatment is feasible. However, owing to the limited number of EPCs and low efficiency of EPC transplantation, vascular injury is not completely repaired in the clinic. Therefore, there is a need to increase the number and improve the morphological traits and function of EPCs.

Vascular endothelial growth factor (VEGF)—a major regulator of vasculogenesis and angiogenesis—is an important angiogenic factor that can regulate vascular regeneration (Millauer et al., 1993). VEGF-165 is a soluble secretory protein that can promote proliferation of EPCs and increase vascular permeability (Chen et al., 2011).

Chemokine receptor 4 (CXCR4, also called CD184) regulates homing of EPCs to vascular injury sites and promotes reendothelialization (Li et al., 2019). Moreover, CXCR4 is the only cognate receptor for chemokine stromal cell derived factor-1 (SDF-1, also called CXCL12) (Dai et al., 2017). SDF-1 is a constitutively expressed and inducible chemokine that plays an important role in proliferation and migration of EPCs (Kawakami et al., 2015). The SDF-1/CXCR4 axis plays a critical guidance function by regulating migration, proliferation, differentiation, and homing of EPCs (Zhang et al., 2018). Previous studies have showed that SDF-1 can not only promote EPCs homing to neovascularization sites, but also cooperate with VEGF to induce EPCs differentiation (Carr et al., 2006). The SDF-1/CXCR4 axis and VEGF can affect each other and enhance their functions by constituting a paracrine loop. SDF-1 can promote the expression of VEGF (Liu et al., 2011), while VEGF can upregulate the expression SDF-1 and promote the expression of CXCR4 by EPCs (Zisa et al., 2011). Thus, increased VEGF expression might increase proliferation of EPCs by promoting the SDF-1/CXCR4 signaling pathway.

With an aim to increase the number and improve the morphological traits and function of EPCs and reduce the cell dose of transplantation, we transducted EPCs with an adenoviral vector carrying VEGF-165. The migration, proliferation, and apoptosis of VEGF-overexpressing EPCs were monitored. Further, we investigated whether VEGF and SDF-1α can act synergistically in EPCs to inhibit vascular stenosis. We established an *in vitro* non-contact co-culture model to compare migration, proliferation, apoptosis, and differentiation capacity of VEGF-transducted EPCs and VSMCs in the presence of exogenous SDF-1α. Finally, we added AMD3100—an SDF-1α receptor antagonist—to block the SDF-1/CXCR4 axis in order to confirm inhibition of vascular stenosis.

## Materials and methods

### Endothelial progenitor cells isolation, culture, and characterization

EPCs were isolated and cultured as previously described (Chen et al., 2010). In brief, bone marrow was extruded from the tibias and femurs of rats. Thereafter, bone marrow mononuclear cells were isolated and plated at a density of 1 × 10^6^ cells/ml in fibronectin pre-coated plates containing endothelial cell basal medium-2 (EBM-2) (Lonza, Switzerland) supplemented with 10% fetal bovine serum (FBS) and 1% treptomycin/penicillin. After 4 days of culture, non-adherent cells were removed by changing the culture medium. Adherent cells were cultured for 7 days and used for experiments. Cell morphology at various stages was observed under an optical microscope to identify EPCs. To confirm their endothelial phenotype, the uptake of DiI-acLDL (Molecular Probes, United States) and FITC-UEA-1 (Sigma, United States) was evaluated. EPCs were washed in PBS, stained with DiI-acLDL (0.02 mg/ml) for 30 min at 37°C, and stained with FITC-UEA-1 (0.01 mg/ml).

Tube formation capacity was assessed using a matrigel assay as previously described (Zhao et al., 2018). EPCs were cultured in EBM-2 with 10% FBS for 14 days and seeded into plates pre-coated with matrigel (Biosciences, United States). The plates were cultured at 37°C with 5% CO_2_ for 12 h. Images of the tube-like structures were acquired in a blinded manner using a light microscope. A minimum of eight fields were examined for each well, and the experiment was repeated with three independent EPC cultures. We tested and analyzed the specific EPC surface markers CD34 FITC (Novus Biologicals, United States), CD133 PE (Abnova, United States), CD31PE (Cell Signaling Technology, United States) and VEGFR-2 PE (Cell Signaling Technology, United States) on days 7 of treatment by laser confocal microscope, as described previously (Chang et al., 2017).

### Animals

All male Sprague-Dawley rats (weighing 250–300 g, 3–4 weeks) were obtained from the Experimental Animal Center of Chongqing Medical University and housed in a suitable environment with ad libitum access water and food. All the animal experiments and procedures were approved by the Institutional Animal Care and Use Committee of Chongqing Medical University and performed under their guidelines.

### Vascular endothelial growth factor overexpression

After 7 days of culture, EPCs were transfected with adenovirus serotype 5 (Ad5) containing enhanced green fluorescent protein gene (EGFP) and VEGF165 gene (abbreviate as Ad5/VEGF) or EGFP as a control (Ad5/EGFP) (Hanheng, China). To obtain optimal transduction efficiency, a serial multiplicity of infection (MOI) was evaluated according to the manufacturer’s manual. After transduction, cells were washed with PBS and incubated with EPC medium for 48 h before subsequent experiments. The EPCs were categorized into three groups: Non-Adv-EPCs, Ad5/EGFP-EPCs, and Ad5/VEGF-EPCs.

### qPCR and western blot analysis

Total RNA was isolated using RNA extraction kit (Takara Bio, China) and qPCR was performed. The following primers were used: VEGF A (sense); 5′- CAA​AGC​CAG​CAC​ATA​GGA​GAG​A-3′, and VEGF B (antisense); 5′-CTA​TCT​TTC​TTT​GGT​CTG​CAT​TCA​C-3′; rat actin A (sense); 5′-CCC​ATC​TAT​GAG​GGT​TAC​GC-3′, and rat actin B (antisense); 5′-TTT​AAT​GTC​ACG​CAC​GAT TTC-3’ (Takara Bio, China). EPC protein was extracted using cell lysis buffer (Beyotime, China) and VEGF protein expression was determined using western blotting. Total cell protein was extracted using RIPA buffer containing phosphatase and protease inhibitors (PMSF) (Beyotime, China). Protein concentration in the supernatant was measured using a BCA protein kit (Boster Bio, China). Protein was separated using SDS-polyacrylamide gel electrophoresis and transferred to a polyvinylidene fluoride (PVDF) (Millipore, United States) membrane. The membrane was incubated overnight at 4°C with primary antibodies against VEGF (1:2,000) and β-actin (1:1,000) (Abcam, United States). The PVDF membranes were washed and incubated with horseradish peroxidase (HRP)-conjugated secondary antibodies (1:5,000) for 30 min. The membranes were washed again, treated with chemiluminescence (ECL) solution (Millipore, United States), and visualized by exposure to film. The expression of each protein relative to β-actin was determined using ImageJ software.

### Vascular smooth muscle cells culture

VSMCs were isolated and cultured as previously described (Zhu et al., 2016). In brief, rat aortic VSMCs were cultured according to ATCC recommendations at 37°C with 5% CO_2_ in DMEM/F-12 supplemented with 10% FBS and 1% treptomycin/penicillin.

### Determination of cell migration, proliferation, apoptosis, and differentiation capacity in co-culture model

The cells in co-culture model were divided into seven groups according to the different treatment as follows: group 1 represents VSMCs and Non-Adv-EPCs; group 2 represents VSMCs and Ad5/EGFP-EPCs; group 3 represents VSMCs and Ad5/VEGF-EPCs; group 4 represents SDF-1α+ VSMCs and Non-Adv-EPCs; group 5 represents SDF-1α+VSMCs and Ad5/EGFP-EPCs; group 6 represents SDF-1α+ VSMCs and Ad5/VEGF-EPCs; group 7 represents SDF-1α+ VSMCs and Ad5/VEGF-EPCs + AMD3100.

The migration assay of VSMCs in co-culture model was performed in 24 well transwell with 8-μm pore polycarbonate membrane insert (Corning, United States). Briefly, 2 × 10^5^ VSMCs in 100 μl serum-free EBM-2 were added into each well of the 24-well insert (upper wells) in triplicate with or without SDF-1 antibody (100 ng/ml) (PeproTech Inc., United States) and incubated for 6 h at 37°C with 5% CO_2_. Thereafter, 2 × 10^5^ EPCs (Non-Adv-EPCs, Ad5/EGFP-EPCs, Ad5/VEGF-EPCs, and Ad5/VEGF-EPCs + AMD3100 (20 μM) (Sigma, United States)) in 500 μl were added into each transwell chamber in triplicate (lower wells). After incubation for 8 h, the migrated VSMCs were fixed with methanol and stained with crystal violet (Beyotime, China) for 10 min. Images of migrated cells were captured using a microscope (100x), and their number was calculated.

Cell proliferation assay of VSMCs in co-culture model was performed in 96 well transwell with 0.4-μm pore polycarbonate membrane insert (Corning, United States) using a CCK-8 kit (Beyotime, China) according to the manufacturer’s instructions. Briefly, 2 × 10^5^ EPCs (Non-Adv-EPCs, Ad5/EGFP-EPCs, Ad5/VEGF-EPCs, and Ad5/VEGF-EPCs + AMD3100) in 75 μl serum-free EBM-2 were added into each well of the 96-well culture plates (upper wells) in triplicate and incubated for 6 h at 37°C with 5% CO_2_. Thereafter, 2 × 10^5^ VSMCs in 235 μl EBM-2 containing 10% FBS were added into each transwell chamber in triplicate (lower wells) with or without SDF-1 antibody (100 ng/ml) and incubated for 48 h at 37°C with 5% CO_2_. Thereafter, VSMCs were incubated with CCK-8 solution for 1 h, the plate was read using a microplate reader at 450 nm.

The apoptosis and differentiation assays of VSMCs in co-culture model were performed in six well transwell with 0.4-μm pore polycarbonate membrane insert (Corning, United States) using flow cytometry (FCM) with propidium iodide (PI), annexin V-FITC dye (Becton, United States) and VSMC surface marker α-SM-actin (Servicebio, China). Briefly, 2 × 10^5^ EPCs (Non-Adv-EPCs, Ad5/EGFP-EPCs, Ad5/VEGF-EPCs, and Ad5/VEGF-EPCs + AMD3100) in 1.5 ml EBM-2 containing 10% FBS were added into each well of the 6-well culture plates (upper wells) in triplicate and incubated for 6 h at 37°C with 5% CO_2_. Thereafter, 2 × 10^5^ VSMCs in 2.5 ml EBM-2 containing 10% FBS were added into each transwell chamber in triplicate (lower wells) with or without SDF-1 antibody (100 ng/ml) and incubated for 48 h at 37°C with 5% CO_2_. After treatment, both dead cells and viable adherent cells were collected and stained with PI and annexin V-FITC or labeled for α-SM-actin. The stained cells were analyzed using FCM.

The migration, proliferation, apoptosis, and differentiation assays of EPCs in co-culture model was the same as described above but with the opposite wells placing the EPCs and VSMCs. Besides, the differentiation assay of EPCs in co-culture model was labeled for CD34 FITC, CD133 PE, CD31PE, and VEGFR-2 PE.

### Statistical analysis

All data are expressed as the mean ± S.E.M. Statistical significance was evaluated by means of Student’s *t*-test or ANOVA. Statistical analysis was performed using SPSS software (version 13.0). A value of *p* < 0.05 was considered statistically significant (*n* = 3 per group; *p < 0.05, **p < 0.01, ***p < 0.001).

## Results

### Identification of endothelial progenitor cells

**FIGURE 1:**
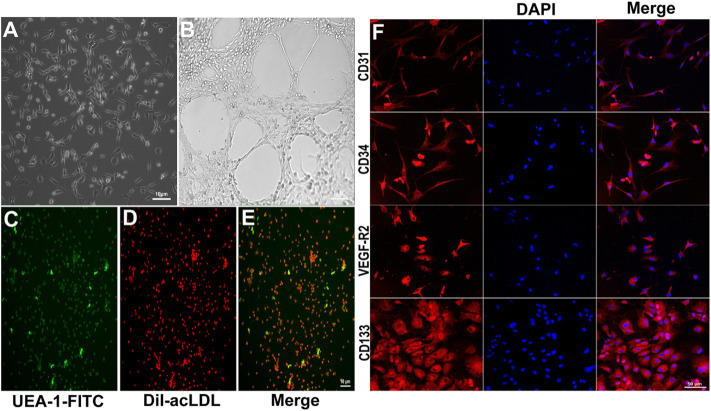
Characterization of cultured EPCs. **(A)** Typical morphological appearance (cluster-like morphology) of EPCs after 7 days of culture. **(B)** Tube formation in matrigel after 14 days culture. The cultured cells were stained with UEA-1-FITC (**C**, green) and DiI-acLDL (**D**, red); merged (**E**, two-color overlay). **(F)** Representative immunofluorescence staining of endothelial cell markers CD31, CD34, VEGF-R2, and CD133 in red imaged using a laser confocal microscope (cell nuclei were counterstained with DAPI, shown in blue. “Merge” represents two-color overlay).

Bone marrow-derived EPCs showed spindle-like morphology and formed cluster-like colonies on day 7 of culture (Figure 1A). The tube formation capacity of EPCs in matrigel was observed on day 14 of culture (Figure 1B). EPCs were stained with UEA-I-FITC and DiI-acLDL (Figures 1C–E). Moreover, EPCs were positively stained for the EPC markers CD31, CD34, VEGF-R2, and CD133 (Figure 1F).

### Transduction of endothelial progenitor cells with vascular endothelial growth factor-containing adenoviral vector

**FIGURE 2:**
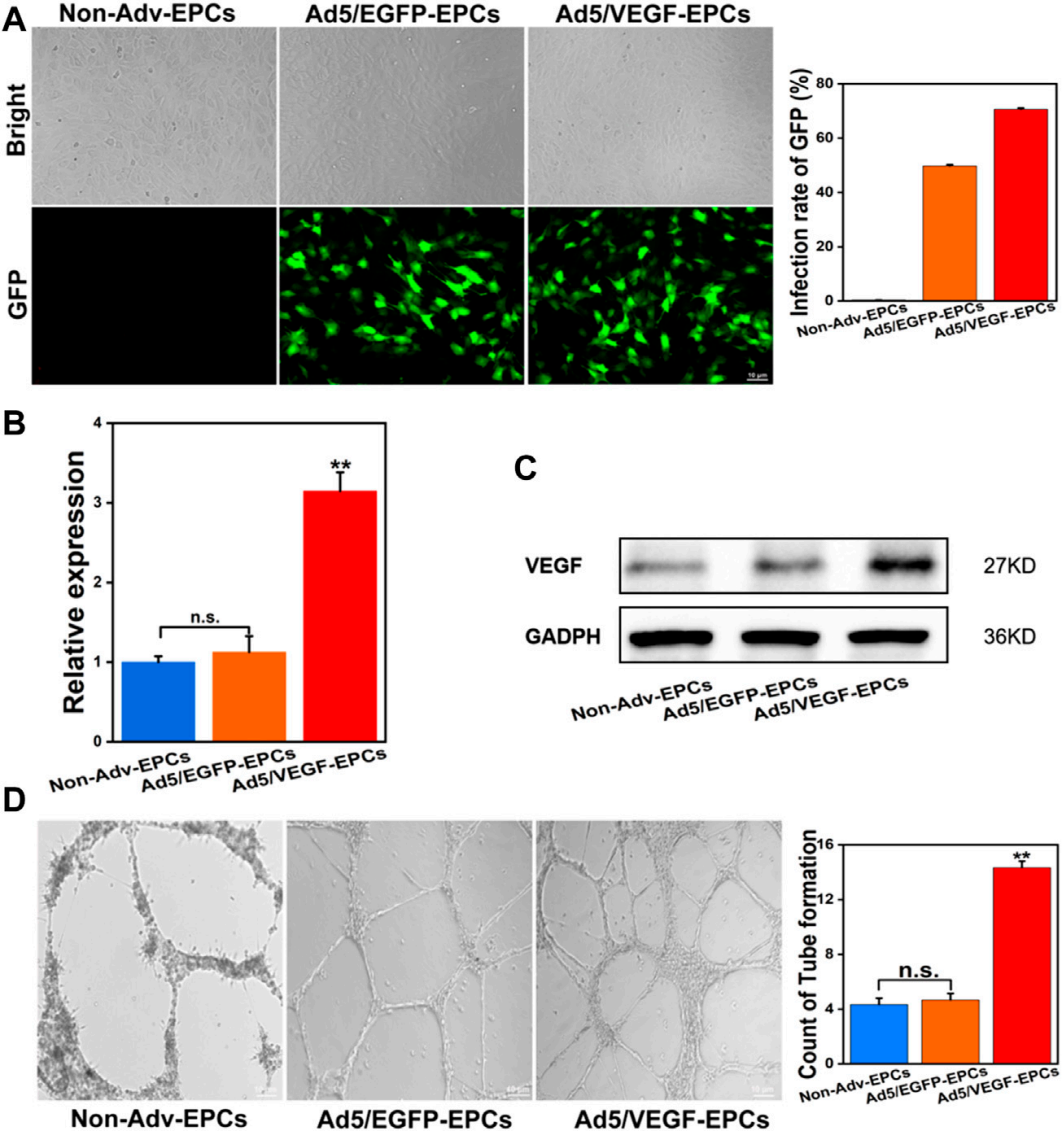
Ad5/VEGF transduction upregulates VEGF expression and improves tube-formation capacity of EPCs. **(A)** Identification of EPCs transducted by Non-Adv, Ad5/EGFP, and Ad5/VEGF by enumerating GFP-positive cells; the graph shows the transduction rate of GFP. **(B)** VEGF mRNA expression was determined using qPCR; actin was used as control. **(C)** VEGF protein expression was assessed by western blotting; GAPDH was used as control. The graph shows a remarkable increase in VEGF expression. **(D)** Tube formation in matrigel on day 14; the number of tubes formed are indicated in the graph.

Rat VEGF165 cDNA was cloned into the Ad5 vector. After the successful completion of preliminary experiments, EPCs were transducted with Ad5/VEGF or Ad/EGFP at a MOI of 50 and incubated in a serum-free culture medium for 1.5 h. At 48 h post-transduction, GFP protein expression was higher in Ad5/VEGF-EPCs and Ad5/EGFP-EPCs than in Non-Adv-EPCs; the transduction rate of GFP was 70.6% (Figure 2A). The transcription and expression of VEGF in EPCs were confirmed by qPCR and western blotting. VEGF mRNA (Figure 2B, *p* < 0.01) and protein (Figure 2C) expression in Ad5/VEGF-EPCs was higher than that in Ad5/EGFP-EPCs and Non-Adv-EPCs. Moreover, tube formation capacity of EPCs was significantly increased (Figure 2D, *p* < 0.01).

### Vascular endothelial growth factor expression improves endothelial progenitor cells function

**FIGURE 3:**
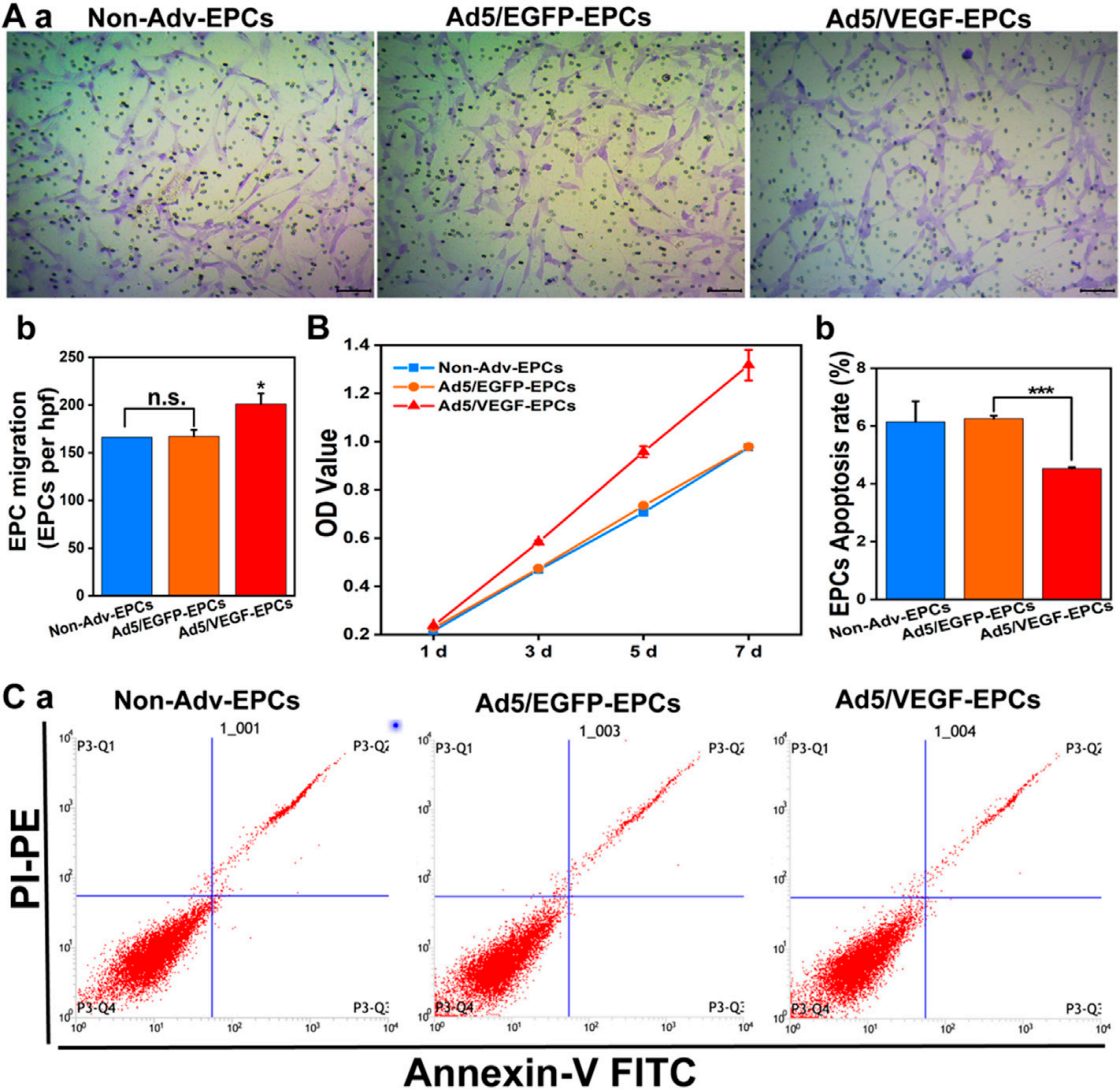
VEGF expression increases proliferation and migration, and decreases apoptosis of EPCs. **(A)** Representative images showing cell migration (a); Bar graph indicates high migration rate of Ad5/VEGF-EPCs (b). **(B)** Proliferation capacity of Ad5/VEGF-EPCs was higher than that of non-Adv-EPCs or Ad5/EGFP-EPCs on days 1, 3, 5, and 7 after transduction. **(C)** Apoptotic rate of EPCs was analyzed by FCM using Annexin V/propidium iodide (PI) staining. Apoptotic cells are defined as Annexin V+/PI- (Quadrant three and 4) (a); (b) Apoptosis rate of Ad5/VEGF-EPCs was significantly lower than that of non-Adv-EPCs or Ad5/EGFP-EPCs.

The migration rate of Ad5/VEGF-EPCs was higher than that of Ad5/EGFP-EPCs and Non-Adv-EPCs (Figure 3A, *p*<0.05). To investigate the proliferation capacity of EPCs after VEGF-induced gene modification, we measured cell viability using CCK-8 on days 1, 3, 5, and 7 post-transduction. The proliferation capacity of Ad5/VEGF-EPCs was higher than that of Ad5/EGFP-EPCs and Non-Adv-EPCs (Figure 3B). Furthermore, FCM analysis showed that the apoptosis rate of Ad5/VEGF-EPCs was lower than that of Ad5/EGFP-EPCs and Non-Adv-EPCs (Figure 3C, *p*<0.001).

### SDF-1α improves endothelial progenitor cells function in the co-culture model

To investigate the effect of SDF-1α on EPC function in the co-culture model, we measured the migration, proliferation, apoptosis, and differentiation capacity of EPCs.

**FIGURE 4:**
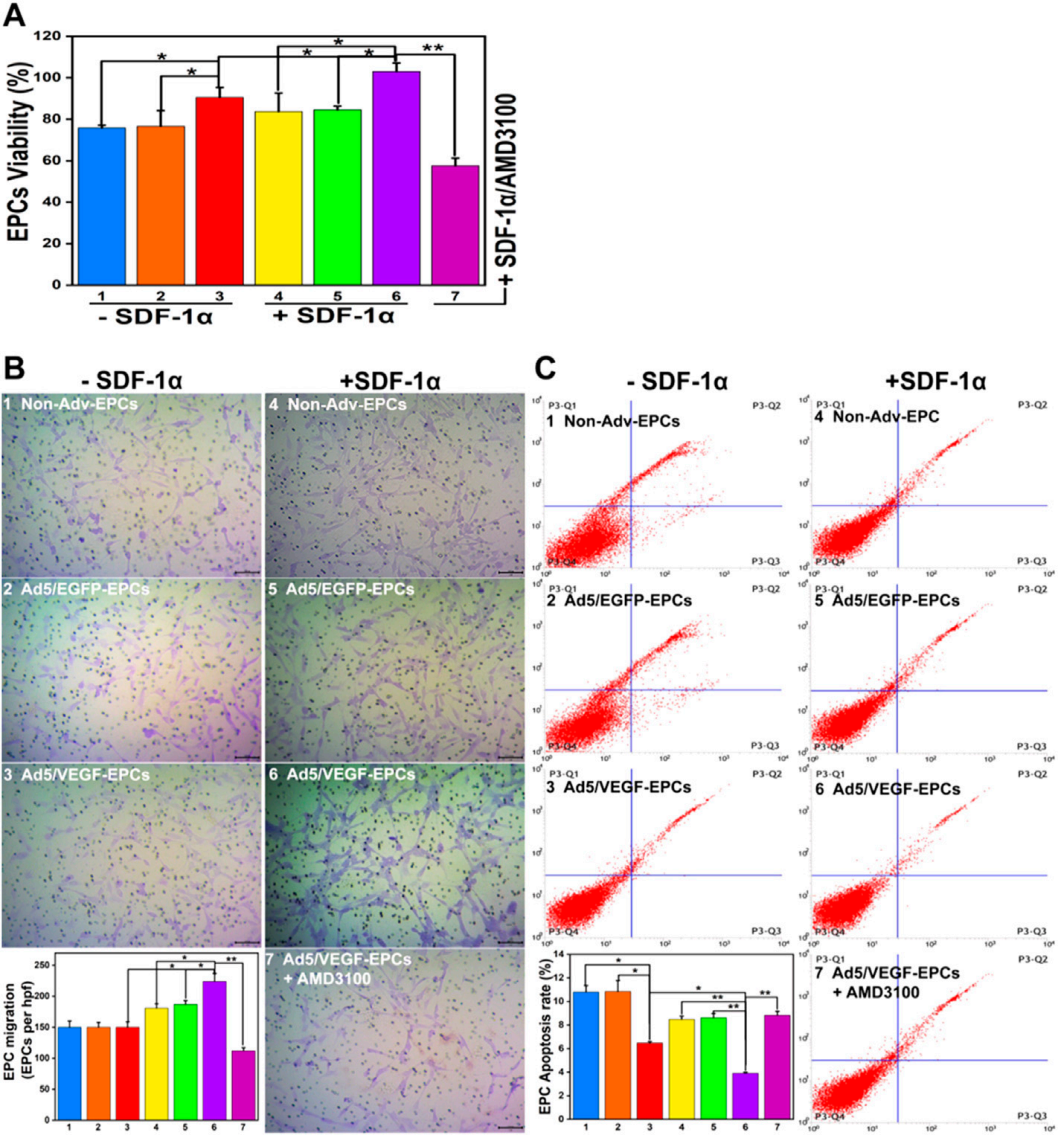
SDF-1α improves EPC function in the co-culture model. **(A)** Proliferation capacity of EPCs in different groups. **(B)** Migration ability of EPCs. **(C)** Apoptosis rate of EPCs (Numbers 1–7 represent VSMCs and Non-Adv-EPCs; VSMCs and Ad5/EGFP-EPCs; VSMCs and Ad5/VEGF-EPCs; SDF-1α+ VSMCs and Non-Adv-EPCs; SDF-1α+ VSMCs and Ad5/EGFP-EPCs; SDF-1α+ VSMCs and Ad5/VEGF-EPCs; SDF-1α+ VSMCs and Ad5/VEGF-EPCs + AMD3100 respectively. *p < 0.05, **p < 0.01, *n* = 3 per group.).

SDF-1α-induced EPCs (groups 4, 5, and 6) exhibited higher proliferation capacity than SDF-1α-non-induced EPCs (groups 1, 2, and 3) (Figure 4A, *p*<0.05). Furthermore, the viability of Ad5/VEGF-EPCs (groups 3 and 6) was significantly higher than that of non-Adv-EPCs (groups 1 and 4) or Ad5/EGFP-EPCs (groups 2 and 5) both in the presence and absence of SDF-1α (Figure 4A, *p*<0.05).

There was no obvious difference in the basal migration capacity among Non-Adv-EPCs (group 1), Ad5/EGFP-EPCs (group 2) and Ad5/VEGF-EPCs (group 3) (Figure 4B). Ad5/VEGF-EPCs (group 6) showed a markedly higher migration response to SDF-1 than non-Adv-EPCs (group 4) or Ad5/EGFP-EPCs (group 5) (Figure 4B, *p*<0.05).

Annexin-PI double staining results showed that the apoptosis rate of SDF-1α-induced EPCs (groups 4, 5, and 6) was lower than SDF-1α-non-induced EPCs (groups 1, 2, and 3) (Figure 4C, *p*<0.05). Moreover, Ad5/VEGF-EPCs (group 6) had a markedly lower apoptotic response to SDF-1 than non-Adv-EPCs (group 4) or Ad5/EGFP-EPCs (group 5) (Figure 4C, *p*<0.01).

FCM results showed positive staining for EPC cell surface markers CD31, CD34, VEGF-R2, and CD133. Moreover, CD31, CD34, and CD133 staining intensity in SDF-1α-induced EPCs was lower than that in SDF-1α-non-induced EPCs (Table 1, *p*<0.05). However, VEGF-R2 staining intensity in SDF-1α-induced EPCs was higher than that in SDF-1α-non-induced EPCs (Table 1, *p*<0.05).

**TABLE 1:** Positive staining of EPC surface markers.

	Non-Adv-EPCs (%)	Ad5/EGFP-EPCs (%)	Ad5/VEGF-EPCs (%)	Non-Adv-EPCs (%)	Ad5/EGFP-EPCs (%)	Ad5/VEGF-EPCs (%)	Ad5/VEGF-EPCs (%)
	+ VSMCs			+ SDF-1α/VSMCs			+SDF1α/VSMCs + AMD3100
CD31	82.72 ± 1.87	80.47 ± 1.52	74.49 ± 1.73^a^	71.39 ± 1.47	69.98 ± 1.05	61.66 ± 1.21^b^	83.17 ± 1.93^a^ ^,^ ^b^
CD34	76.65 ± 0.97	75.77 ± 0.91	71.80 ± 0.87^a^	61.00 ± 0.62	58.10 ± 0.60	51.79 ± 0.53 ^**c**^	86.91 ± 1.98^a^ ^,^ ^b^
VEGF-R2	93.05 ± 0.74	94.21 ± 0.95	95.41 ± 1.28^a^	98.10 ± 0.73	98.32 ± 0.092	98.37 ± 1.02	91.86 ± 0.64^a^ ^,^ ^b^
CD133	3.22 ± 0.22	3.15 ± 0.32	2.92 ± 0.44^a^	1.67 ± 0.34	1.64 ± 0.07	1.07 ± 0.14 ^**c**^	4.89 ± 0.16^a^ ^,^ ^b^

Note: *n* = 3 per group; data is presented as the mean ± SD.

a *p* < 0.05 vs. (non-Adv-EPCs, or Ad5/EGFP-EPCs) + VSMCs.

b *p* < 0.05 vs. (non-Adv-EPCs, or Ad5/EGFP-EPCs) + SDF-1α/VSMCs.

### SDF-1α inhibits vascular smooth muscle cells function in the co-culture model

To investigate the effect of SDF-1α on VSMC function in the co-culture model, we measured the migration, proliferation, apoptosis, and differentiation capacity of VSMCs.

**FIGURE 5:**
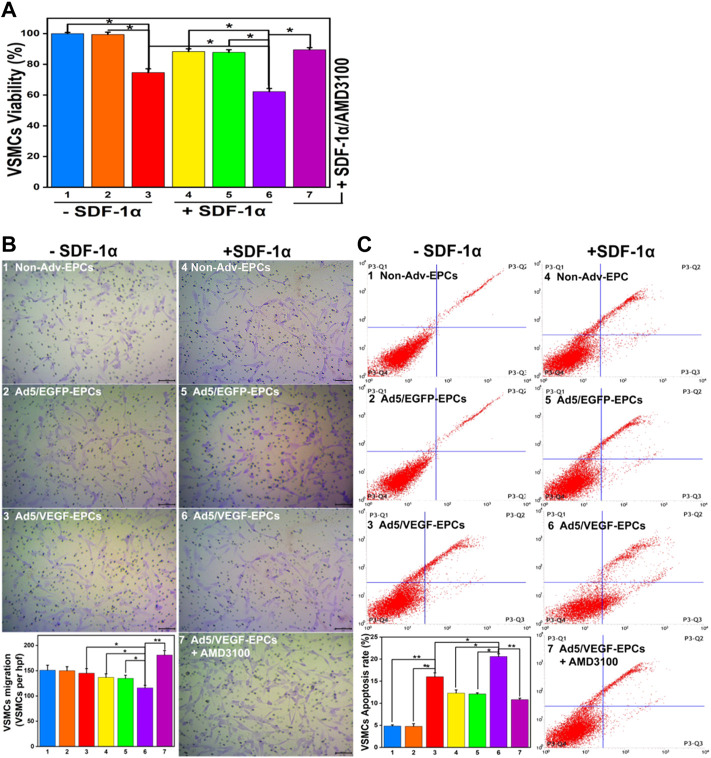
SDF-1α decrease VSMC function in the co-culture model. **(A)** Proliferation capacity of VSMCs in different groups. **(B)** Migration ability of VSMCs. **(C)** Apoptosis rate of VSMCs (Numbers 1–7 represent VSMCs and Non-Adv-EPCs; VSMCs and Ad5/EGFP-EPCs; VSMCs and Ad5/VEGF-EPCs; SDF-1α+VSMCs and Non-Adv-EPCs; SDF-1α+ VSMCs and Ad5/EGFP-EPCs; SDF-1α+VSMCs and Ad5/VEGF-EPCs; SDF-1α+VSMCs and Ad5/VEGF-EPCs + AMD3100 respectively. *p < 0.05, **p < 0.01, *n* = 3 per group.).

SDF-1α-induced VSMCs (groups 4, 5, and 6) exhibited lower proliferation capacity than SDF-1α-non-induced EPCs (groups 1, 2, and 3) (Figure 5A, *p*<0.05). Furthermore, the viability of VSMCs in the Ad5/VEGF-EPC (groups 3 and 6) was lower than that of VSMCs in the non-Adv-EPC (groups 1 and 4) or Ad5/EGFP-EPC (groups 2 and 5) both in the presence and absence of SDF-1α (Figure 5A, *p*<0.05).

There was no observable difference in the basal migration capacity among Non-Adv-EPCs (group 1), Ad5/EGFP-EPCs (group 2) and Ad5/VEGF-EPCs (group 3) (Figure 5B). Ad5/VEGF-EPCs (group 6) displayed a lower migration response to SDF-1α than non-Adv-EPCs (group 4) or Ad5/EGFP-EPCs (group 5) (Figure 5B, *p*<0.05).

Annexin-PI double staining results showed that the apoptosis rate of SDF-1α-induced VSMCs (groups 4, 5, and 6) was observably higher than that of SDF-1α-non-induced VSMCs (groups 1,2, and 3) (Figure 5C, *p*<0.05). Moreover, Ad5/VEGF-EPCs (group 6) had a markedly higher apoptosis response to SDF-1α than non-Adv-EPCs (group 4) or Ad5/EGFP-EPCs (group 5) (Figure 5C, *p*<0.01).

FCM results showed positive staining of VSMCs for α-SM-actin. α-SM-actin staining intensity in SDF-1α-induced VSMCs was observably higher than in SDF-1α-non-induced VSMCs (Table 2, *p*<0.05).

**TABLE 2:** Positive staining of VSMC surface markers.

	Non-Adv-EPCs (%)	Ad5/EGFP-EPCs (%)	Ad5/VEGF-EPCs (%)	Non-Adv-EPCs (%)	Ad5/EGFP-EPCs (%)	Ad5/VEGF-EPCs (%)	Ad5/VEGF-EPCs (%)
				+ SDF-1α			+ SDF-1α+AMD3100
α-SM-actin	35.46 ± 0.51	40.42 ± 0.83	53.21 ± 1.06^a^	64.48 ± 0.74	76.60 ± 1.21	83.18 ± 0.92^b^	86.52 ± 0.65^a^ ^,^ ^b^

Note: *n* = 3 per group; data is presented as mean ± SD.

a *p* < 0.05 vs. non-Adv-EPCs, or Ad5/EGFP-EPCs.

b *p* < 0.05 vs. (non-Adv-EPCs, or Ad5/EGFP-EPCs) + SDF-1α/VSMCs.

### AMD3100 pre-treatment affects endothelial progenitor cells and vascular smooth muscle cells function

To further elucidate the involvement of VEGF and the SDF-1/CXCR4 axis in the functional behavior of EPCs and VSMCs, we treated EPCs and VSMCs with the SDF-1α receptor antagonist AMD3100. Compared with the Ad5/VEGF-EPCs (group 6), the proliferation, migration, and differentiation capacity of EPCs was significantly inhibited upon AMD3100 pre-treatment, and the apoptosis rate was increased (Figures 4, 5, Tables 1, 2, *p*<0.01). However, the proliferation, migration, and differentiation capacity of VSMCs was significantly increased upon AMD3100 pre-treatment when compared with the Ad5/VEGF-EPCs (group 6), and the apoptosis rate was decreased (Figures 4, 5; Tables 1, 2; *p*<0.01).

## Discussion

The rapid development of intravascular technology has allowed endovascular treatment of cardiovascular diseases. However, vascular stenosis remains a challenge for vascular biologists and cardiologists. Vascular stenosis is associated with endothelial cell apoptosis, macrophage adhesion and invasion, and smooth muscle migration and growth (Iqbal et al., 2013). Therefore, accelerating reendothelialization after arterial injury becomes important for vascular repair.

It has been reported that EPCs are the most advantageous source of seed cells for vascular repair due to their ability maintain vascular endothelial function and integrity (Landmesser et al., 2004). EPCs are recruited by cytokines and inflammatory factors to the intimal lining of the injured region and stimulate migration and proliferation of the neighboring endothelial cells by secreting angiogenic growth factors (Rodrigues et al., 2013). EPCs play a key role in the treatment of vascular stenosis; however, endogenous EPCs are limited in number and are insufficient to meet the need of clinical applications. Therefore, *in vitro* isolation and culture of EPCs are essential to meet their clinical application requirements. In this study, we successfully isolated numerous EPCs from rat bone marrow with high proliferative capacity.

VEGF binds to cell-surface receptors and plays an important role in vascular repair (Hill et al., 2003). VEGF can induce proliferation, migration, and differentiation of EPCs after vascular injury, in a mechanism involving the MAPK, AKT, and Notch pathways, as well as Cx43-mediated gap junctions (Asai et al., 2013). In this study, we transducted VEGF-expressing adenoviral vector into EPCs using an optimum MOI of 50. qPCR and western blotting results showed high expression of VEGF in VEGF-expressing EPCs. We also measured VEGF mRNA and protein expression in EPCs that were not transducted with VEGF-containing vector to demonstrate low expression of endogenous VEGF in EPCs. These results further validate the need to upregulate VEGF in EPCs through gene transduction. Consequently, the tube formation, proliferation, and migration capacity of EPCs was increased upon transduction with VEGF; however, the apoptosis rate was decreased.

Previous studies have demonstrated that the SDF-1/CXCR4 axis plays an important role in EPC migration and homing (Kawakami et al., 2015). The binding of SDF-1 to CXCR4 can regulate EPC function by mediating calcium overload, activating the PI3K-AKT-NF-κB axis, and phosphorylating MAPK (Liang et al., 2016). SDF-1 not only promotes mobilization of EPCs to neovascularization sites, but also induces their differentiation into mature endothelial cells when co-expressed with VEGF (Liu et al., 2011). Furthermore, the SDF-1/CXCR4 axis and VEGF can synergistically form paracrine loops to enhance their biological functions (Zisa et al., 2011). SDF-1 can increase CXCR4 expression, activate the SDF-1/CXCR4 axis, and elevate VEGF levels in EPCs. On the other hand, VEGF can upregulate SDF-1 expression to sensitize EPCs to SDF-1 (Kucia et al., 2004).

Herein, we investigated the synergistic effect of VEGF and SDF-1α in EPCs and VSMCs. We established a non-contact co-culture model to determine the migration, proliferation, apoptosis, and differentiation capacity of EPCs and VSMCs in the presence or absence of SDF-1α. Our results indicate that overexpression of VEGF increases proliferation, migration, and differentiation of EPCs in the presence of SDF-1α, but decreases apoptosis. However, the opposite effect was observed in VSMCs. These results suggest that VEGF-overexpressing EPCs can be used as a promising therapy for vascular stenosis.

Studies have showed that the recruitment and integration of EPCs to damaged sites is not the only mechanism for the repair of vascular endothelial injury; in fact, a paracrine mechanism also performs similar function (Bhatwadekar et al., 2010). In this mechanism, cytokines including prostaglandin I2 (PGI2), nitric oxide (NO), calcitonin gene-related peptide (CGRP), and vascular growth factors such as VEGF and SDF-1 are secreted. These cytokines can block the synthesis of DNA and total proteins of VSMCs to inhibit its pathological proliferation and phenotypic transformation (Segal et al., 2006). Further, EPCs can inhibit the proliferation of angiotensin II-induced VSMCs and decreased the expression of proto-oncogenes such as c-myc and c-fos (Fang et al., 2011). One study has suggested that EPCs express Jagged1 to inhibit phenotypic transformation of VSMCs (Zhang et al., 2014). Thus, EPCs can promote paracrine signaling in the presence of exogenous SDF-1α to inhibit proliferation and migration, and promote apoptosis of VSMCs.

There are some limitations of our study. First, SDF-1α showed concentration dependence on EPCs (Sheng et al., 2018). We did not use varying concentrations of SDF-1α to test the effect of the SDF-1/CXCR4 axis on EPCs. Second, the effect of VEGF gene transfer on disease state EPCs was not investigated. Therefore, it is necessary to understand the signaling pathways that affecting proliferation, migration, differentiation, and apoptosis of EPCs and VSMCs. Third, studies have revealed that SDF-1 also can bind and signal through the CXCR7 receptor, even stronger binding affinity for SDF-1 than CXCR4 in T lymphocytes (Balabanian et al., 2005), subsequently, SDF-1/CXCR4/CXCR7 signaling axis was proved in many biological processes, such as immune and nervous systems, hematopoiesis, and cardiovascular disease (Guyon, 2014; Zhang et al., 2018). We should make some deeper research to explore the role of SDF-1/CXCR4/CXCR7 signaling axis between EPC and VSMCs.

In conclusion, both VEGF and SDF-1α can promote EPC function *in vitro*. Moreover, both proteins work the synergistically to promote EPC function and inhibit VSMC function. This therapeutic strategy can be used for the prevention of vascular stenosis.

## Data Availability

The original contributions presented in the study are included in the article/supplementary material, further inquiries can be directed to the corresponding author.
